# Histology and scanning electron microscopy of the tubal tonsil of goats

**DOI:** 10.14202/vetworld.2015.1011-1014

**Published:** 2015-08-25

**Authors:** V. R. Indu, K. M. Lucy, J. J. Chungath, N. Ashok, S. Maya

**Affiliations:** 1 Department of Veterinary Anatomy and Histology, College of Veterinary and Animal Sciences, Mannuthy, Kerala Veterinary and Animal Sciences University, Kerala, India; 2 Department of Veterinary Anatomy and Histology, College of Veterinary and Animal Sciences, Pookode, Kerala Veterinary and Animal Sciences University, Kerala, India

**Keywords:** goats, histology, tubal tonsil

## Abstract

**Aim:**

To observe the light and scanning electron microscopy (SEM) of the caprine tubal tonsil.

**Materials and Methods:**

The study was conducted on six crossbred male goats of 6 months of age. From the median sections of the head, tissue pieces from the nasopharynx around the auditory tube were collected and fixed for histology and SEM.

**Results:**

Tonsillar lymphoid tissue was located in the nasopharynx ventral to the auditory tube opening in the lateral wall of the pharynx. The height of the surface epithelium of the tubal tonsil measured 80.17±1.08 µm and was a pseudostratified ciliated columnar type with basal, supporting, and goblet cells. Above the dome of lymphoid nodules, the epithelium was modified into a follicle associated epithelium (FAE), also called lympho-epithelium or reticular epithelium and was characterized by the absence of goblet cells and cilia, reduced number of cell layers, and a large number of lymphoid cells due to interrupted basement membrane. The height of FAE was smaller than that of the surface epithelium and measured 34.33±0.92 µm. The surface of tubal tonsil showed folds and invaginations, which formed crypts. The lamina propria-submucosa underneath the epithelium was formed by the meshwork of reticular and, thin and loose collagen fibers with dome-like accumulation of lymphoid nodules. In the secondary lymphoid nodules, a corona, parafollicular area, and interfnodular area were observed. The average number of lymphoid nodules counted per field under low power magnification of microscope was 1.17±0.17, and the internodular distance was 34.00±4.37 µm. The mean diameter of lymphoid nodules was 566.67±11.45 µm and the lymphocyte count per nodule was 14741.67±174.36. The number of plasma cells counted per field under low power was 44.38±2.90 below the surface epithelium. The tubal tonsil was not encapsulated. In SEM, the surface epithelium of the tubal tonsils presented ciliated cells, microvillus (MV) cells, and goblet cells. The region of FAE possessed Type-I and Type-II MV cells and microfold (M) cells in between.

**Conclusion:**

It was concluded that the tubal tonsils were well developed in goats, which might serve as a means of protection against the spread of infection to the middle ear cavity.

## Introduction

The tonsils represented a first line of defense against ingested and inhaled foreign antigens [1]. The tubal tonsils seen in the nasopharynx around the pharyngeal opening of the Eustachian tube is an intermediate type of tonsil [2] and constituted a part of nasal associated lymphoid tissue and a component of Waldeyer’s ring [3,4]. M-cells were reported to be present in the nasopharyngeal and tubal tonsils of the horse [3]. These specialized M-cells were involved in active transfer of soluble and particulate matter across the epithelium [5], and thus tonsils served as effector organs of local, systemic, and mucosal adaptive immunity to the airborne and alimentary antigens [6].

Tissues from tonsils form most valuable clinical material for diagnostic investigations of infectious diseases and are used for preclinical screening of scrapie in goats. Due to its numerous advantages, practice of intranasal immunization in domestic animals is on a steady rise. The pharyngeal and tubal tonsils are the main targets for nasal vaccines and are of prime importance for the diagnosis of certain infectious diseases [7].

Although the tubal tonsil has been reported in humans, ruminants, pigs, and horse, information on its structure is very few [8]. Hence, the present work was undertaken to observe the light and scanning electron microscopy (SEM) of the caprine tubal tonsil.

## Materials and Methods

### Ethical approval

This research work was carried out after getting approval from the Research Committee and Institutional Animal Ethics Committee.

### Materials studied and staining

The study was conducted on six crossbred male goats of 6 months of age, brought for slaughter from the University Sheep and Goat Farm, Mannuthy. From the median sections of the head, tissue pieces from the nasopharynx around the auditory tube were collected and fixed in 10% neutral buffered formalin. The materials were processed routinely to obtain 5-6 µm thick serial paraffin sections. The sections were stained using hematoxylin and eosin [9], Gomori’s rapid one-step trichrome method for collagen fibers [9], Verhoeff’s method for elastic fibers [9], Gordon and Sweet’s method for reticular fibers [10], and Unna’s method for mast cells [9].

For SEM, samples of tubal tonsils were fixed in 2.5% glutaraldehyde in 0.1 M phosphate buffer saline (pH 7.2) for 24 h at 4°C and post fixed in 2% aqueous osmium tetroxide for 4 h. Thereafter, the samples were processed and scanned under SEM (SEM-model: JEOL-JSM 5600) at required magnifications atRuska Labs, College of Veterinary Science, Sri Venkateswara Veterinary University, Rajendra Nagar, Hyderabad, Andhra Pradesh.

## Results and Discussion

### Tonsillar surface epithelium

**Figure-1:**
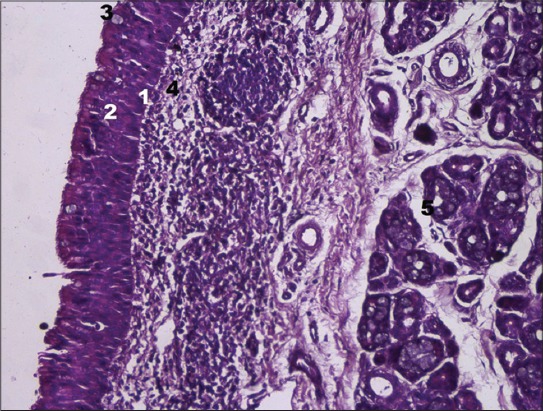
Cross section of tubal tonsil of goat showing surface epithelium (H and E, ×400). (1) Basal cell, (2) supporting cell, (3) goblet cell, (4) lamina propria-submucosa, and (5) glandular acini.

Surface epithelium of the tubal tonsil consisted of pseudostratified ciliated columnar cells. It presented 6-10 rows and revealed nuclei of three types of cells, *viz*. basal, supporting, and goblet cells (Figure-1). In basal cells, the nuclei were elongated and vertically placed with a distinct nucleolus, which was eccentric in position. The supporting cells were of two varieties namely, Type-I cells with dark round to oval nuclei situated more superficially in the epithelium and Type-II cells with large sized but less basophilic nuclei distributed irregularly throughout the epithelium. The nuclei of the goblet cells were located basally because of the presence of large mucous granules above it. Cytoplasm of all the cell types was finely granular and eosinophilic. Similar observations were made in sheep [11].

### Follicle associated epithelium (FAE)

**Figure-2:**
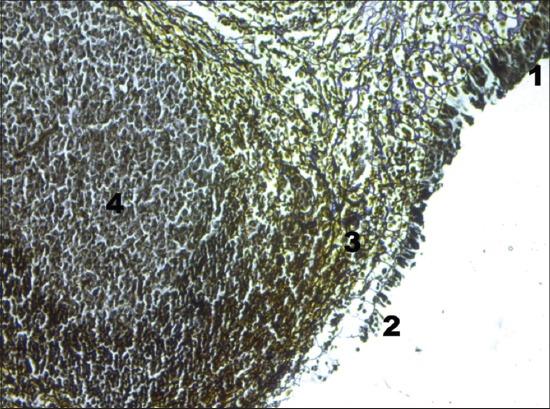
Cross section of tubal tonsil showing follicle associated epithelium (Gordon and Sweet silver impregnation method, ×400). (1) Surface epithelium, (2) follicle-associated epithelium, (3) lymphocytes in meshwork of reticular fibers, and (4) lymphoid nodule.

The surface of tubal tonsil showed folds and invaginations, which formed crypts. The epithelium mounting over the dome of lymphoid nodules was modified into FAE, also called lympho-epithelium or reticular epithelium and was characterized by the absence of goblet cells, reduced number of cell layers and absence of cilia, and a large number of lymphoid cells due to interrupted basement membrane (Figure-2). The height of reticular epithelium measured lesser than that of the surface epithelium These observations confirmed the reports in tubal tonsils of horse [3] and sheep [11] and in soft palate tonsils of sheep [12]. The epithelial cells in FAE produced a secretory component and polymeric immunoglobulin (Ig) receptor, which stabilized and transported secretory IgA to the mucosal surface [13]. The FAE helped in intake of antigens, transportation of immunocytes, and protection of mucosal surfaces [14].

### Propria-submucosa

The lamina propria-submucosa underneath the epithelium was formed by a meshwork of reticular fibers and, thin and loose collagen fibers with dome-like accumulation of lymphoid nodules. The collagen fiber layer underneath the epithelium was thin and loose compared with those in the oropharyngeal and laryngopharyngeal tonsils [15].

**Figure-3:**
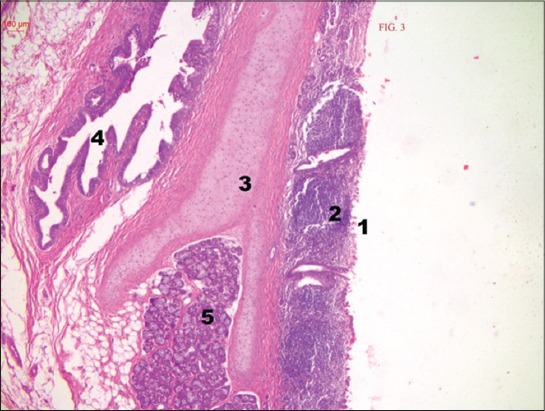
Cross section of tubal tonsil showing lymphoid tissue ventral to the opening of the auditory tube. (H and E, ×40). (1) Lateral pharyngeal wall, (2) lymphoid tissue, (3) cartilage, (4) auditory tube opening, and (5) glandular acini.

Tonsillar lymphoid tissue was found mainly ventral to the auditory tube opening in the lateral wall of the nasopharynx (Figure-3). It consisted of secondary lymphoid nodules and diffused lymphoid tissue arranged as tonsillar follicles or crypto-lymphatic units and tonsillar nodules in the superficial lamina propria similar to the reports in horse [16]. However, it was reported earlier that in ruminants the tubal tonsil was of non-follicular type [17].

**Figure-4:**
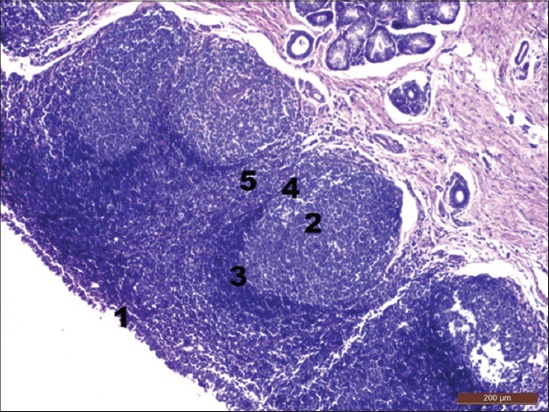
Cross section of tubal tonsil showing lymphoid nodules (6 months) (H and E, ×100). (1) Follicle-associated epithelium, (2) lymphoid nodule, (3) corona, (4) parafollicular area, and (5) internodular area.

In the secondary lymphoid nodules, a corona, parafollicular area, and internodular area were observed (Figure-4). In a few lymphoid nodules, the corona containing dark staining small lymphocytes was seen toward the epithelium. The high endothelial venules were distributed more toward the internodular area. Large number of small, medium, and large sized lymphocytes, macrophages, and plasma cells were seen within the nodules. These observations are in accordance with the reports in sheep [12], goat [18], and camel [19].

The average number of the lymphoid nodules per field was 1.17±0.17 with an internodular distance of 34.00±4.37 µm. The average diameter of lymphoid nodules was 566.67±11.45 µm and lymphocyte count per nodule was 14741.67±174.36. In the present study, the lymphocyte count per nodule in tubal tonsils was more than that of the tonsil of soft palate (1372.39±260.02) and paraepiglottic tonsils (10833.33±557.77) but was lesser as compared to the palatine (28826.54±236.25) and pharyngeal tonsils (32233.23±324.24). The number of plasma cells counted per field under low power magnification of microscope was 44.38±2.90 below the surface epithelium. Similar reports on the micrometry of lymphatic tissue in tubal tonsils of goats are not available for comparison.

Below the nodules in the deeper lamina propria, dense arrangement of collagen and elastic fibers was seen near the cartilage and in between the clusters of glandular acini. Small nerve bundles and adipose tissue were also observed. The tubal tonsil was not encapsulated. These observations concurred with the reports in the tubal tonsil of sheep [12].

### SEM

**Figure-5:**
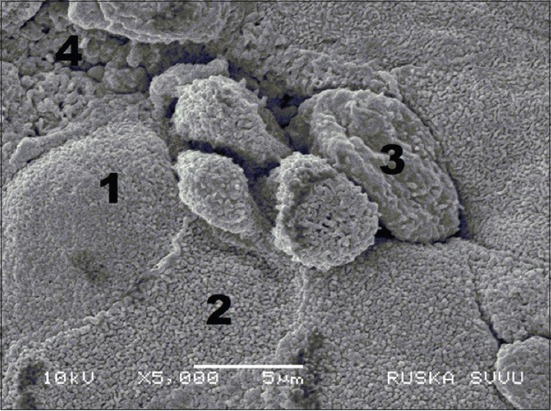
Section of tubal tonsil showing follicle associated epithelium with disrupted columnar cells (6 months) (Scanning electron microscopy, ×1000). (1) Type-I microvillus (MV) cells, (2) Type-II MV cells, (3) microfold cells, and (4) lymphocytes seen between disrupted columnar cells.

In the SEM, surface epithelium of the tubal tonsils of 6-month-old goats presented cells with a dense mat of cilia, microvillus (MV) cells, and goblet cells. The region of FAE possessed Type-I MV cells having homogeneous small-sized MV and Type-II MV cells with large-sized MV. In between them, some cells with very small-sized MV or microfold were identified as M-cells. In some areas, the FAE presented interrupted columnar cells with MV and numerous lymphoid cells in between (Figure-5). These observations are in accordance with the reports in horse [3], sheep [11], and camel [20]. The lack of goblet cells in the FAE reduced the thickness of the epithelium and modified the composition of mucus layer over FAE [21].

## Conclusion

The tubal tonsils in crossbred goats were seen ventral to the auditory tube opening in the lateral wall of the nasopharynx. The tonsil was not encapsulated and possessed crypts. The well-developed tubal tonsil in these goats may serve as a means of protection against the spread of infection to the middle ear cavity.

## Authors’ Contributions

VRI collected references, samples, and performed laboratory investigation. KML monitored the study. JJC, NA, and SM helped in the laboratory investigations, drafted and revised the manuscript. All authors read and approved the final manuscript.
